# A Case of Pan-TRK Positive Dermatofibrosarcoma Protuberans Located on the Nose

**DOI:** 10.7759/cureus.54215

**Published:** 2024-02-14

**Authors:** Romane Teshima, Taiyo Hitaka, Hitomi Sugino, Etsuko Okada, Yu Sawada

**Affiliations:** 1 Dermatology, University of Occupational and Environmental Health, Kitakyushu, JPN

**Keywords:** case report, pan-trk staining, ntrk fusion gene, face, s: dermatofibrosarcoma protuberans

## Abstract

Dermatofibrosarcoma protuberans (DFSP) is a rare and infiltrative soft tissue tumor. Our report details a distinctive case of DFSP with pan-TRK positivity in the right nasal dorsum of a 46-year-old female. Histological analysis identified *NTRK* fusion gene involvement in this patient, detectable through pan-TRK immunostaining. The case underscores the significance of comprehensive management for pan-TRK-positive DFSP in challenging facial locations, indicating the potential efficacy of TRK inhibitors.

## Introduction

Dermatofibrosarcoma protuberans (DFSP) is a rare soft tissue tumor characterized by slow, infiltrative growth that mostly arises in the trunk and extremities [1]. Recently, NTRK rearrangement neoplasm is a specific type of cancer involving mutations in the *NTRK* gene, which codes for the neurotrophic tyrosine kinase receptor and plays a role in cell growth and differentiation [2]. The rearrangement of this gene typically leads to the loss of normal control, potentially causing abnormal proliferation of cancer cells. Although recently updated research findings indicate that the fusion of the *COL1A1* gene with the *PDGFB* gene is a distinct characteristic of DFSP [3], and this gene rearrangement is also identified in a few cases of DFSP [4], the detailed clinical characteristics remain unclear. Here, we report a case of DFSP with pan-TRK positivity.

## Case presentation

**Figure 1:**
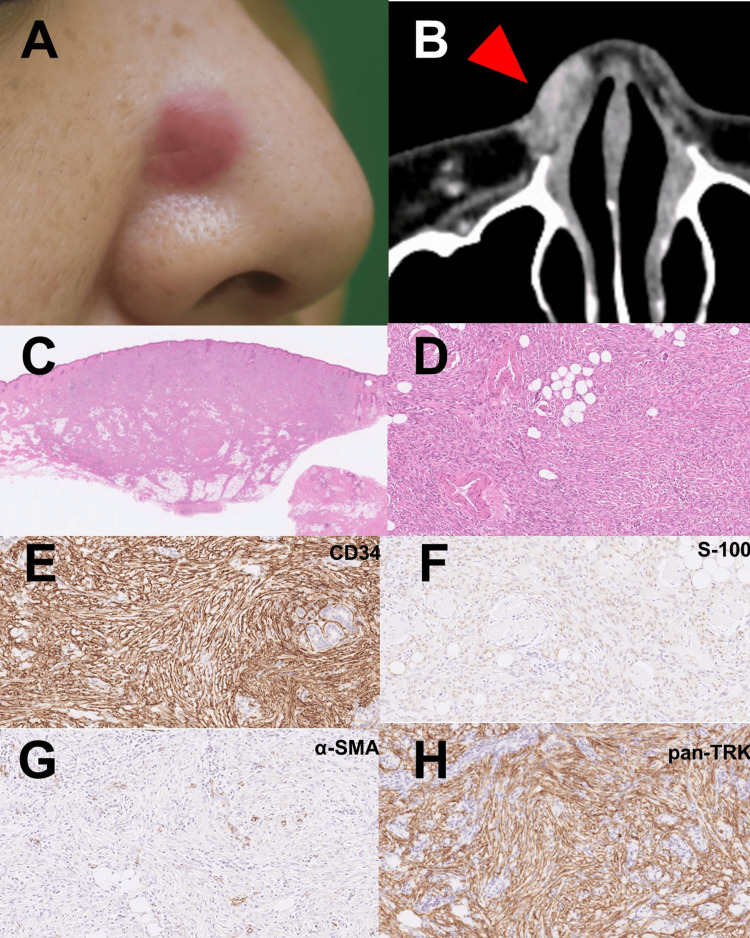
Clinical manifestations, computed cosmography imaging, and histological analysis. (A) Clinical manifestation of the tumor; (B) CT showing the tumor on the nose (Red arrow); (C, D) H&E staining, low magnification view (C) and high magnification view of the tumor (D); (E-H) Immunohistochemical staining for CD34 (E), S-100 (F), α-SMA (G), and pan-TRK (H).

A 46-year-old female presented with a 15×10 mm reddish infiltrative nodule on the right nasal dorsum (Figure 1A). It was discovered by her one year prior to the presentation and gradually developed. A systemic computed tomography imaging showed no distant metastasis. A histological examination taken from the nodule revealed spindle-shaped tumor cells proliferating in a storiform pattern, with positive immunostaining for CD34 and vimentin, and negative staining for S-100. Based on these findings, a diagnosis of DFSP was established. Based on the computed cosmography imaging showing the deeper invasion of the tumor (Figure 1B), a wide local excision was performed. Additionally, the right lateral nasal cartilage, right greater alar cartilage, and nasal mucosa were entirely excised. The wound was covered and closed using a free forearm flap.

Histopathological analysis of the excised whole specimen revealed mildly atypical spindle cells arranged in a storiform pattern within the true dermis to subcutaneous tissue (Figures 1C, 1D). Subsequent immunohistochemical staining revealed diffuse positivity for CD34 and pan-TRK, while S-100 and αSMA showed partial positivity (Figures 1E-1H). Evaluation of the surgical margins indicated a proximity to the deep nasal cartilage. Therefore, an additional excision, involving the complete removal of the right lateral nasal cartilage and the lower end of the nasal bone, resulted in the removal of residual tumor. Regular outpatient visits were conducted at intervals of one to three months to examine for any local recurrence and there has been no recurrence of the tumor one year after the surgical resection.

## Discussion

The rarity of DFSP in facial locations, constituting less than 10% of cases, was highlighted. Our facial DFSP case is exceptionally rare, highlighting the difficulties in ensuring necessary margins given the functional and aesthetic significance of facial structures. Although we could not conduct it in this patient, Mohs micrographic surgery might be helpful in minimum surgical resection of the tumor. Based on preoperative imaging examinations, we conducted excision following the standard resection for DFSP. However, due to incomplete removal of the tumor at the initial excision, we decided to proceed with wide excision. Effective management requires a comprehensive approach, combining preoperative imaging and histopathological assessment.

The *NTRK* fusion gene arises from the abnormal fusion of the *NTRK* gene, responsible for tropomyosin receptor kinase involved in nerve cell differentiation and maintenance, with other genes such as *ETV6*, *LMNA*, and *TPM3* due to chromosomal translocations [5,6]. Recent studies suggest the potential efficacy of TRK inhibitors against tumors carrying *NTRK* fusion genes, making this gene rearrangement detectable through pan-TRK immunostaining [4,7]. In DFSP, 15% of cases exhibit pan-TRK positivity, and ongoing data collection is expected to uncover clinical differences related to this phenomenon [4]. Our case showed aggressive invasion into deeper skin layers, making surgical resection difficult. Therefore, caution may be warranted in such a case of pan-TRK-positive DFSP. On the contrary, since the detailed characteristics of pan-TRK-positive DFSP remain unclear, it is uncertain that this is unique to pan-TRK-positive tumors as shown in this case. We need to accumulate cases and analyze whether this is a specific clinical feature of pan-TRK-positive DFSP for the future.

This case emphasizes the importance of precise preoperative planning, thorough pathological evaluation, and consideration of emerging molecular targets for optimal management of pan-TRK-positive DFSP in challenging facial locations.

## Conclusions

This case underscores the rarity and clinical challenges posed by DFSP with pan-TRK positivity, especially in facial locations. The successful management of the presented case involved wide local excision due to the general non-use of Mohs surgery for DFSP in our country and careful consideration of aesthetic and functional factors. The *NTRK* fusion gene's involvement in DFSP, with a number of cases exhibiting pan-TRK positivity, highlights potential treatment avenues with TRK inhibitors. Although CT imaging was conducted to assess the tumor's invasion into the nasal bone and systemic metastasis, MRI imaging will be the best choice for delineating the DFSP three-dimensional structure. Precise preoperative planning, thorough pathological evaluation, and an understanding of emerging molecular targets are crucial for effective management. Despite the rarity of facial DFSP cases, their complexity necessitates a comprehensive approach for optimal outcomes.

## References

[1] Zhou X, Sun D, Liu Y (2020). Dermatofibrosarcoma protuberans: our 10-year experience on 80 patients. J Dermatolog Treat.

[2] Siozopoulou V, Smits E, De Winne K, Marcq E, Pauwels P (2021). NTRK fusions in sarcomas: diagnostic challenges and clinical aspects. Diagnostics (Basel).

[3] O'Brien KP, Seroussi E, Dal Cin P (1998). Various regions within the alpha-helical domain of the COL1A1 gene are fused to the second exon of the PDGFB gene in dermatofibrosarcomas and giant-cell fibroblastomas. Genes Chromosomes Cancer.

[4] Hung YP, Fletcher CD, Hornick JL (2018). Evaluation of pan-TRK immunohistochemistry in infantile fibrosarcoma, lipofibromatosis-like neural tumour and histological mimics. Histopathology.

[5] Doebele RC, Davis LE, Vaishnavi A (2015). An oncogenic NTRK fusion in a patient with soft-tissue sarcoma with response to the tropomyosin-related kinase inhibitor LOXO-101. Cancer Discov.

[6] Drilon A, Li G, Dogan S (2016). What hides behind the MASC: clinical response and acquired resistance to entrectinib after ETV6-NTRK3 identification in a mammary analogue secretory carcinoma (MASC). Ann Oncol.

[7] Hechtman JF, Benayed R, Hyman DM (2017). Pan-TRK immunohistochemistry is an efficient and reliable screen for the detection of NTRK fusions. Am J Surg Pathol.

